# A bypass flow model to study endothelial cell mechanotransduction across diverse flow environments

**DOI:** 10.1016/j.mtbio.2024.101121

**Published:** 2024-06-13

**Authors:** Zhuotao Xiao, Rudmer J. Postma, Anton Jan van Zonneveld, Bernard M. van den Berg, Wendy M.P.J. Sol, Nicholas A. White, Huybert J.F. van de Stadt, Asad Mirza, Jun Wen, Roel Bijkerk, Joris I. Rotmans

**Affiliations:** a Department of Internal Medicine (Nephrology) and the Einthoven Laboratory for Vascular and Regenerative Medicine, Leiden University Medical Center, Leiden, 2333, ZA, Netherlands; b Department of Nephrology, The First Affiliated Hospital of Soochow University, Suzhou, 215000, China; c Department of BioMechanical Engineering, Delft University of Technology, Delft, 2628, CN, Netherlands; d Department of Medical Technology, Design & Prototyping, Leiden University Medical Center, Leiden, 2333, ZA, Netherlands; e Department of Biomedical Engineering, Florida International University, Miami, FL, 33199, United States; f Department of Computer Science and Technology, Southwest University of Science and Technology, Mianyang, 621010, China

**Keywords:** Disturbed flow, *In vitro* model, Vascular bifurcation, Computational fluid dynamics, High-content screening

## Abstract

Disturbed flow is one of the pathological initiators of endothelial dysfunction in intimal hyperplasia (IH) which is commonly seen in vascular bypass grafts, and arteriovenous fistulas. Various *in vitro* disease models have been designed to simulate the hemodynamic conditions found in the vasculature. Nonetheless, prior investigations have encountered challenges in establishing a robust disturbed flow model, primarily attributed to the complex bifurcated geometries and distinctive flow dynamics. In the present study, we aim to address this gap by introducing an *in vitro* bypass flow model capable of inducing disturbed flow and other hemodynamics patterns through a pulsatile flow in the same model. To assess the model's validity, we employed computational fluid dynamics (CFD) to simulate hemodynamics and compared the morphology and functions of human umbilical venous endothelial cells (HUVECs) under disturbed flow conditions to those in physiological flow or stagnant conditions. CFD analysis revealed the generation of disturbed flow within the model, pinpointing the specific location in the channel where the effects of disturbed flow were observed.

High-content screening, a single-cell morphological profile assessment, demonstrated that HUVECs in the disturbed flow area exhibited random orientation, and morphological features were significantly distinct compared to cells in the physiological flow or stagnant condition after a two days of flow exposure. Furthermore, HUVECs exposed to disturbed flow underwent extensive remodeling of the adherens junctions and expressed higher levels of endothelial cell activation markers compared to other hemodynamic conditions.

In conclusion, our *in vitro* bypass flow model provides a robust platform for investigating the associations between disturbed flow pattern and vascular diseases.

## Graphical abstract

**Figure:**
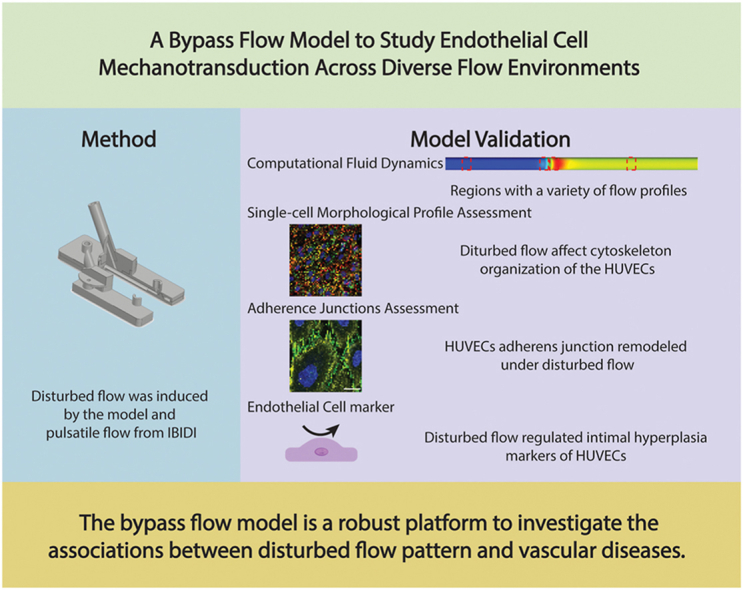

## 1 Introduction

Intimal hyperplasia (IH) is the primary pathological causes of vascular disease, vascular bypass, or hemodialysis vascular access failure, which commonly develop at the vascular bifurcation and its host vessels, causing narrowing of the vessel lumen [1]. The vascular bifurcation and bypass structure after surgery, together with the native pulsatile blood flow pattern, result in a disturbed flow at these location which is marked by low and oscillatory wall shear stress (WSS) [2,3]. Time-averaged wall shear stress (TAWSS), which is used to estimate the average value of WSS throughout one cardiac cycle due to the non-uniform distribution of WSS, is notably low in the disturbed flow region [4]. The oscillatory shear index (OSI), a marker quantifying the degree of flow reversal within a single cardiac cycle, and relative residence time (RRT) which represents the residence time of blood near endothelial cells are relatively high in disturbed flow location [5,6]. Besides the low and oscillatory WSS, a WSS multi-directionality parameter named transverse WSS (TransWSS) is also increasing in the disturbed flow area [7]. Various studies revealed that the abnormalities in these parameters are related to EC dysfunction and the development of vascular disease in the disturbed flow location [3,6,[8], [9], [10]].

The process by which altered hemodynamics trigger IH by ECs dysfunction has been extensively studied [11,12]. Adherens junctions are crucial cell-cell junctions that maintain cell-cell adhesion, and regulate endothelial cell barrier permeability, and thus contribute to the maintenance of vascular homeostasis. Adherens junctions and cell morphology are sensitive to hemodynamic changes [13]. In a physiological laminar flow pattern, ECs elongate and align with the direction of flow and express mature adherens junction because of the uniform WSS which is characterized by unidirectional WSS vector and a high WSS value. ECs also exhibit an anti-inflammatory phenotype by up-regulating the mechanosensitive transcription factor named Krüppel-like factor 2 (KLF2) [[14], [15], [16]]. Conversely, disturbed flow with oscillatory and low WSS, is a pathological flow pattern, resulting in ECs loosing parallel alignment and elongation, and remodeling of cell-junctions. Contrary to laminar flow conditions, KLF2 expression is downregulated while inflammatory factors are upregulated in disturbed flow areas [[17], [18], [19], [20]]. In addition, studies have shown that high RRT and OSI can predict a spectrum of pathological alterations and their occurrence in a disturbed flow area [9].

*In-vitro* disease models are essential for studying pathophysiology and drug development, as it is difficult to obtain disturbed flow affected samples from patients, and animal disturbed flow models cannot fully replace human studies [21,22]. In previous studies, disturbed flow was simulated using oscillatory flow with low WSS [[23], [24], [25]], despite the similarities between these two flow patterns, oscillatory flow is an axial, periodic, and regular flow, hence it cannot entirely replace the non-uniformity and true complexity of disturbed flow. Cone-and-plate viscometer and orbital shaker platforms are also frequently employed to model the multidirectional flow and the non-uniform WSS in disturbed flow [26,27]. Nevertheless, these devices are unable to accurately replicate the complex hemodynamics in bifurcation and disregard the 3D tubular shape of vessels. They also fall short in simulating the native EC microenvironment as the compliant extracellular matrix is absent in these models. Microfluidic devices have become a valuable tool for studying the effects of fluid dynamics on cells because of the precise fluid control, incorporation of relevant extracellular matrices (ECM), and ability to create custom designs [28]. Microfluidic devices commonly have dimensions at micrometer scale, however, the diameters of coronary arteries and arteriovenous bypass grafts range from 2 to 8 mm [[29], [30], [31]], making the hemodynamics of these anatomical structures more complex than can be achieved in most microfluidic devices.

To recapitulate the complex hemodynamics at vascular branches and bypasses, we aimed to develop a millimeter-scale tubular cell culturing model that can recreate disturbed flow with pulsatile flow and branching structure, incorporating both primary ECs and native collagen-based ECM. We employed computational fluid dynamics (CFD) to predict the position of disturbed flow areas in our model to which ECs are directly exposed. We validated our model and CFD by quantifying the effects of disturbed flow on EC morphology, cell-cell adherens junctions, and EC activation markers. Cost-effectiveness, modularity, and standardization are also taken into consideration during the model design process to expand application domains.

## 2 Materials and methods

### 2.1 Bypass flow model design

**Fig. 1:**
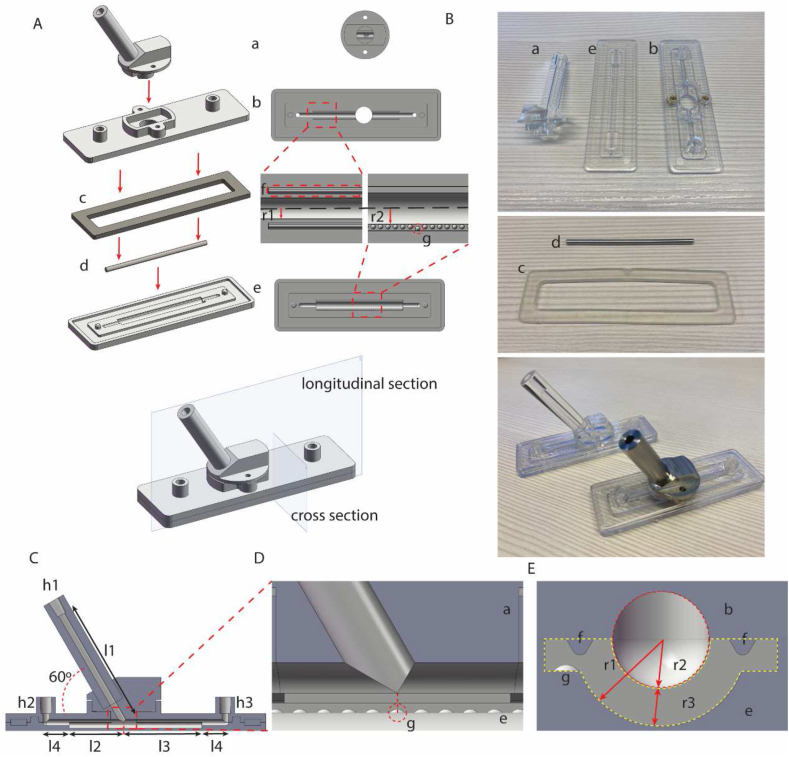
In vitro bypass flow model design. (A) SolidWorks design of the model, a branch; b cover; c gasket; d steel rod; e plate. Bottom view of the cover, main channel (r1 = 1.25 mm); f ridge. Upper view of the plate, main channel (r2 = 2.25 mm); g convex points, origin point (red circle). (B) Overview of the disassembled parts and assembled product. (C) Longitudinal section view of the model after assembling the 3 primary parts, h: Luer connector, h1 branch Luer, h2 left Luer, h3 right Luer; l1 = 45 mm; l2 = 20.55 mm; l3 = 29.45 mm; l4 = 10 mm; angle = 60°. (D) Longitudinal section view of the intersection area. Origin point (red circle) was directly below the intersection of the branch channel and cover channel. (E) Cross section view of the model after assembling the 3 primary parts, r1 = 2.25 mm; r2 = 1.25 mm; r3 = 1 mm; main channel (red circle); cavity for collagen (yellow area). (For interpretation of the references to colour in this figure legend, the reader is referred to the Web version of this article.)

The model was designed as a hollow branching structure in SolidWorks 2020 (SolidWorks Corp., USA), and manufactured from polystyrene through plastic injection molding (Arburg 320 KS 700-250, NL). The three primary parts were a branch, a plate, and a cover. Additional accessories included a silicone waterproof gasket and a stainless-steel rod (r2 = 1.25 mm). They could be assembled as Fig. 1A, and Fig. 1B shows the product of our model. The bottom view of the cover reveals a 70 mm long main channel (r2 = 1.25 mm) laying in the center of this part with two 0.4 mm high ridges on both sides (Fig. 1A). In the center of the plate, there was a 50 mm long main channel (r1 = 2.25 mm), and it extended 1 cm (l4 = 1 cm) as extension channels (r2 = 1.25 mm) on both sides of axial direction for placing rod. Two platforms were also designed in the radial direction of the main channel, and convex points spaced 1 mm apart were located on one platform (Fig. 1A). Fig. 1C and D shows the longitudinal section of the model after assembling the 3 primary parts, the angle between the branch and the cover was 60°, at the end of each channel, a female Luer locker could be connected to an Ibidi pump system (Ibidi GmbH, Martinsried, Germany) or plugged by a 1 ml syringe. The origin point (Fig. 1D) which was directly below the intersection of the branch channel (r2 = 1.25 mm) and cover channel separated the main channel into two parts (l2 = 29.45 mm, l3 = 20.55 mm). Fig. 1E shows the cross-section view of the model, the main channel (red circle) was formed by the cover and the plate, after placing the rod on the extension channel, a 50 mm long cavity (thickness r3 = 1 mm) for collagen was left under the rod (yellow area).

### 2.2 Cell culture

Primary Human Umbilical Vein Endothelial Cells (HUVECs; Leiden University Medical Center, LUMC) were isolated from umbilical cords obtained from the department of obstetrics at the LUMC, as previously described [32]. Cells were maintained in completed Endothelial Cell Growth Medium 2 (EGM-2) (Promo Cell), supplemented with 50 U/ml of penicillin/streptomycin (Gibco) and Supplemented Mix (Promo Cell). Cells were grown on freshly coated plates with 1 % gelatin (Merck) in PBS until passages 3–4. Cells were re-suspended and seeded into the bypass flow model after reaching 80 % confluency. Before seeding, the bypass flow model was soaked in 70 % ethanol overnight and disinfected by UV for 30 min, while accessories were sterilized in autoclave.

### 2.3 Design of perfusion model

The bypass flow model was given a constant or pulsatile flow via the IBIDI flow system. To provide a continuous flow of 0.5 mL/min (0.17 cm/s), the pump pressure was set to 3 mbar and switched to 55 mbar to provide a 15 mL/min (5.1 cm/s) pulsatile flow (1 s/cycle). The flow volume was determined by measuring the fluid collected in a syringe per minute based on the manufacturer's instruction. The flow waveform was validated by comparing the pressure waveform detected by a pressure sensor at the inlet of the model and the inlet pressure waveform recorded in CFD (Fig. S1). The completed medium was used as the perfusion medium which was mixed with 0.7 mg/ml Xanthan gum (Sigma-Aldrich) to increase the viscosity [19,33], and the medium was refreshed every day.

### 2.4 Computational fluid dynamics

CFD validated the bypass flow and predicted the hemodynamics in the channel. Initial preprocessing was conducted using the ANSYS ICEM CFD 2022 R1 software (ANSYS Inc., Pittsburgh, PA, USA) to create non-structural meshes. The setting for the boundary layer was configured to five layers, with the detailed mesh representation depicted in Fig. S2. Subsequently, mesh sensitivity study was executed to guarantee the stability of computational outcomes, Table S1. Ultimately, the number of elements for the model was determined as approximately 1,595,107.

The medium was thought to be uniform, isothermal, incompressible Newtonian fluid. The governing equations were shown as follows:

(1) $$\begin{array}{c} \frac{\rho \partial \vec{u}}{\partial t}+\rho \left(\vec{u}∙\nabla \right)\vec{u}+\nabla p-\mu \Delta \vec{u}=0 \end{array}$$

(2) $$\begin{array}{c} \nabla ∙\vec{u}=0 \end{array}$$

Where $\vec{u}$ is the flow velocity vector, p is the pressure, ρ is the medium density of 1020 kg/m^3^, μ is the dynamic viscosity 0.0035kg/m∙s**.**

A fully developed pulsatile flow profile was applied at the inlet. The inlet flow waveform was based on the flow pattern provided by the Ibidi system, the peak flow velocity was 5.1 cm/s. At the outlet, the traction-free outflow boundary condition was applied. No-slip boundary condition was applied to all walls and a rigid wall model was assumed.

The governing equations were solved numerically by a finite-volume method and the CFD code, ANSYS Fluent (Version 2022 R1, Ansys, Inc, USA), using a fully implicit second-order backward Euler differencing scheme. The convergence criterion (a normalized residual, obtained based on the imbalance in the linearized system of discrete equations) was set to 10^−8^ in this study. The time-step size was taken to be 0.01 s after time-step independence study, Table S2, and the results were recorded at the end of each time-step. To eliminate the start-up effects of transient flow, the computation was carried out for three periods. The third period results are presented.

CFD-post was analyzed by MATLAB 2020 (MathWorks, USA) and plotted by Tecplot 360 EX 2020 RI (Tecplot, Inc, USA)

The calculation method of TAWSS, OSI, RRT, TransWSS is shown as follows:

(3) $$\begin{array}{c} \text{TAWSS}=\frac{1}{T}\int_{0}^{T}\left|\vec{\tau_{w}}\right|\text{dt} \end{array}$$

(4) $$\begin{array}{c} \text{OSI}=0.5\left[1-\frac{\left|\int_{0}^{T}\vec{\tau_{w}}\text{dt}\right|}{\int_{0}^{T}\left|\vec{\tau_{w}}\right|\text{dt}}\right] \end{array}$$

(5) $$\begin{array}{c} \text{RRT}=\frac{1}{\left(1-2∙\text{OSI}\right)∙\text{TAWSS}} \end{array}$$

(6) $$\text{TransWSS}=\frac{1}{T}\int_{0}^{T}\left|\vec{\tau_{w}}∙\left(n\times \frac{\int_{0}^{T}\vec{\tau_{w}}dt}{\left|\int_{0}^{T}\vec{\tau_{w}}dt\right|}\;\right)\right|dt$$

Where T is a cardiac cycle of pulsation and $\vec{\tau_{w}}$ is an instantaneous wall shear stress vector, n is the normal vector to the surface.

### 2.5 Immunofluorescent staining

Collagen lining of the main channel was removed 48 h after the start of the flow experiment. Regions of interest, as predicted by CFD, were identified by the markers on the model and isolated. Samples were fixated by submersion in 4 % PFA for 10min, following permeabilization by incubating with 0.3 % triton-X100 in PBS for 10mins, blocked by 5 % bovine serum albumin (BSA) (Sigma-Aldrich) in PBS for 30mins. Samples were incubated with primary antibody solutions against Von Willebrand Factor or VE-cadherin (1:500, Polyclonal Rabbit Anti-Human Von Willebrand Factor, Dako; 1:200, Purified Mouse Anti-Human CD144, BD; 1:200, Alexa Fluor 647 Mouse Anti-Human CD144) at 4 °C overnight, washed with PBS 3 times, and then incubated with appropriate secondary antibodies (Alexa Fluor 488 goat anti-mouse IgG1 and Alexa Fluor 647 goat anti-rabbit IgG, Invitrogen, respectively) in presence of phalloidin-Rhodamine (Invitrogen) and Hoechst (Molecular Probes) in PBS with 0.5 % BSA for 1 h at room temperature. Images were taken by confocal microscope Dragonfly-200 (Andor Technology, Belfast, UK) using 40X (NA 1.33) or 63X (NA 1.52) objective lenses.

### 2.6 High-content screening

Single-cell morphological profiles were extracted from the confocal microscopy images as reported previously [34]. Briefly, CellProfiler (version 4, BROAD Institute, Boston, MA) was used to segment individual nuclei from DAPI-channel images by local threshold, followed by applying watershed segmentation. After debris was filtered out by a lower threshold for nuclei size, individual nuclei were used as seeds to identify cells by Voronoi-Based segmentation using the VE-cadherin signal to determine cell borders. Image-based features such as texture, moment, intensity, distribution, colocalization, and shape were extracted [35,36]. Data analysis of the extracted features was performed in R (version 4.3.1). First, the dataset was normalized by Z-score, following this the dataset was reduced in dimension by factor analysis capturing at least 80 % of total variance [37]. Linear discriminant analysis (LDA) was then used for further dimensional reduction and classification to interpret the discriminations between each groups [38].

### 2.7 Cell junction analysis

The types of adherens junction were referred to the study which distinguished them into stable adherens junctions (AJs) and focal adherens junctions (FAJs) [39]. AJs were defined as junctions which are aligned by parallel F-actin, while FAJs are connected to F-actin bundles and orientated perpendicularly of VE-cadherin. Spatial overlap of VE-cadherin and F-actin was colocalized in ImageJ-win64 (Fiji) [40]. Difference between AJs and FAJs was quantified as ratio FAJ length over total junction (AJ + FAJ) length. For each of the locations of interest predicted by CFD, twenty-five cells were analyzed.

### 2.8 Quantitative reverse transcription-polymerase chain reaction

Collagen with cells was cut into four samples based on the locations predicted by CFD, total RNAs were extracted by RNeasy Mini Kit (Qiagen) followed with the manual. RNA was then reverse-transcribed into cDNA by a M-MLV Reverse Transcriptase Kit (Promega, Singapore), while qPCR was performed with SYBR Select Master Mix (Waltham, MA, USA; Applied Biosystems) in Bio-Rad CFX384 Touch™ Real-Time PCR Detection System. Primers were designed based on the NCBI reference sequence database (Table S3).

### 2.9 Statistical analysis

All data shown in violin graphs were presented as median (interquartile range, IQR). The data shown in bar graphs were presented as means ± SEM, while n represents the number of biological independent experiments. Groups were compared by one-way ANOVA and P < 0.05 was considered as statistically significant.

## 3 Results

### 3.1 HUVECs seeding in the bypass flow model

**Fig. 2:**
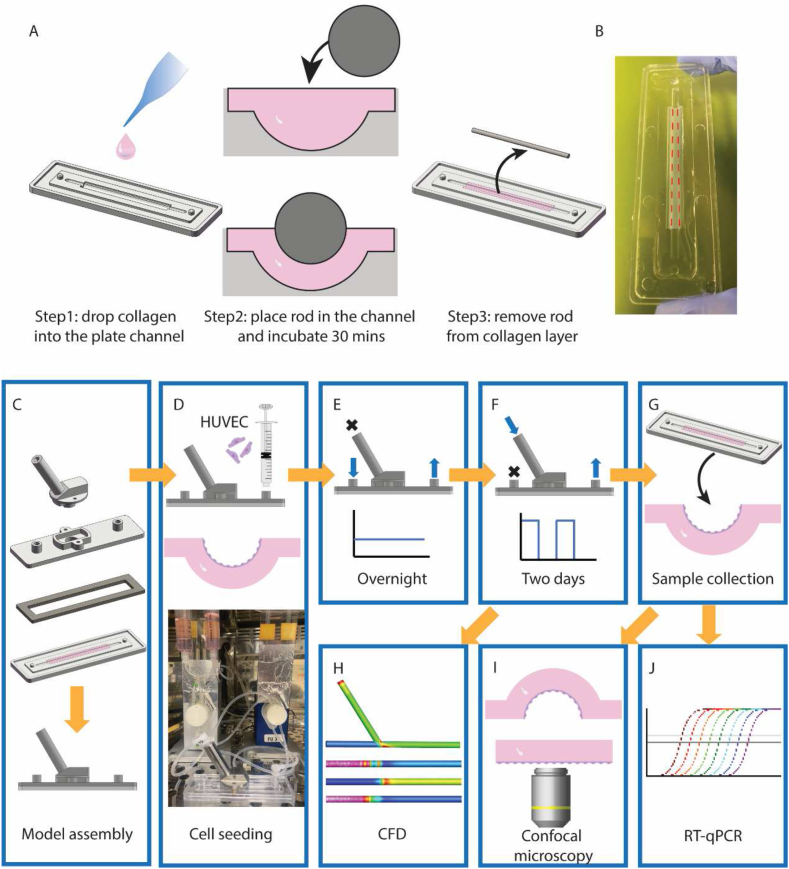
Schematic diagram of flow experiment. (A) Collagen layer was made by three steps: 1 drop collagen into the plate channel; 2 place the rod on the extension channel of the plate, and force collagen remaining in the cavity under the rod for 30 min 37° incubation; 3 remove rod from the collagen layer. (B) Collagen layer with semi round channel was laid in the center of the plate, red dash line showed the edges of the collagen channel. (C) Assemble the model. (D) Inject HUVECs to the surface of the collagen layer from the Luer, link the model to the IBIDI system after HUVECs attaching the collagen surface. (E) HUVECs were cultured overnight in constant flow. (F) HUVECs were cultured in disturbed flow for two days. (G) Collagen was removed from the plate after the flow experiment for either immunofluorescent staining or RNA extraction. (H) CFD simulated hemodynamics in the model and predicted flow pattern in different locations. (I) Flattened the collagen surface with HUVECs by attaching this side to a glass coverslip for confocal microscopy. (J) RT-qPCR detected gene expression from different locations. Portions of the figure utilized images from Servier Medical Art (Servier; https://smart.servier.com/), licensed under Creative Commons Attribution 4.0 Unported License. (For interpretation of the references to colour in this figure legend, the reader is referred to the Web version of this article.)

On the day of the experiment, 300 μl of 4 % rat tail collagen was made by mixing collagen (Cultrex 3-D Culture Matrix Rat Collagen I, R&D system), PBS, and sodium bicarbonate at a ratio of 1:1:8 on ice. The collagen was immediately injected into the channel of the plate, and the steel rod was mounted in the extension channel. The collagen was forced to form a 1 mm thick semi-round layer in the cavity shown in Fig. 1E. The plate with collagen was incubated in a humidified incubator at 37 °C for 30 min to promote collagen polymerization. After collagen gelation, the steel rod was easily removed from the collagen (Fig. 2A) and left a collagen layer with a semi round channel (r1 = 1.25 mm) in the plate (Fig. 2B). Then, the complete model was assembled with the plate, cover, and branch (Fig. 2C). To prevent fluid leakage, clamps and a silicone gasket were employed on the model. Meanwhile, a suspension of HUVECs (5.5 X 10^5 cells/ml) was prepared and injected into the model via the Luer with a syringe, followed by a 45-min incubation at 37 °C for cells attaching the collagen. Finally, the model was linked to the IBIDI system (Fig. 2D). To promote forming a confluent monolayer on the collagen surface, the HUVECs were exposed to a constant left to right flow (0.17 cm/s, 0.5 ml/min) overnight by plugging the branch Luer and linking IBIDI system to the two horizontal Luer (Fig. 2E). The next day, the left Luer was plugged, and the tube was moved to the branch Luer providing a pulsatile flow with peak flow at 5.1 cm/s (15 ml/min, 1s/cycle) from the IBIDI system (Fig. 2F). Based on the flow setting in IBIDI system and model configuration, we predicted the flow pattern and dynamic distribution in the model by CFD (Fig. 2H). After 2 days of flow experiment, the model was disassembled, and collagen was removed for either immunofluorescent staining or RNA extraction (Fig. 2I and J). To achieve a high image quality of confocal microscopy, we flattened the collagen surface with HUVECs by attaching this side to a glass coverslip.

### 3.2 Computational fluid dynamics validated and predicted the hemodynamics in four areas of the model

**Fig. 3:**
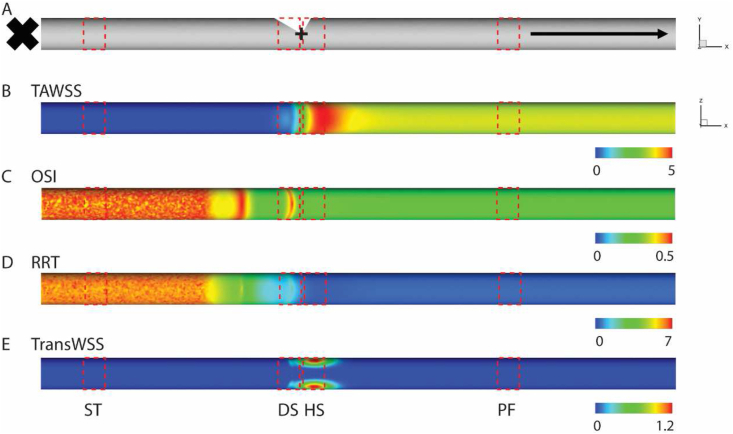
Hemodynamics in the channel of bypass flow model. (A) Four locations, ST, DS, HS, PF, on the channel wall were divided based on the hemodynamic property, width of red rectangle = 2 mm. Distribution of TAWSS, dyne/cm^2^ (B), OSI (C), Log (RRT), 1/Pa (D), TransWSS, dyne/cm^2^ (E) on the channel wall. (For interpretation of the references to colour in this figure legend, the reader is referred to the Web version of this article.)

The entire collagen extract was partitioned into four sections based on the hemodynamic characteristics displayed on the channel wall (Fig. 3A, Table 1). The CFD revealed that our model induced an intensive unsteady flow on the surface of collagen opposite to the intersection. The area 2 mm on the left side of the origin point was defined as the disturbed flow area (DS) where the significant low TAWSS and high OSI, RRT, TransWSS were observed (Fig. 3B). The eddies could also be observed at the flow switching timepoint in DS (Supplement video 1, Fig. S3). it should be noted that the Reynolds number in our model was thirty-nine corresponding to a laminar flow. However, we still defined the flow pattern in this area as disturbed to maintain consistency with previous studies. The area 2 mm on the right side of the origin point was defined as the higher WSS area (HS). TAWSS in HS was the highest among the four areas, while OSI and RRT were the lowest, TransWSS was as high as the DS, recirculation eddies were still present during switching flow (Fig. 3B–Supplement video 1, Fig. S3). The hemodynamics executed a regular and stable pulsatile flow pattern in the long range between the HS and right Luer, and we define this area as physiological flow area (PF) to distinguish it from other location. In this location, the flow direction was unidirectional, OSI, RRT and TransWSS were low, and the TAWSS distribution was uniform (Fig. 3B–Supplement video 2, Fig. S3). Due to the plugged Luer on the left, the region between the left Luer and DS had the lowest TAWSS as the flow velocity was close to zero, while the values of OSI and RRT were high because the fluid in this region was still subject to pulsatile pressure (Fig. 3B). We defined this area as stagnation area (ST). Though the areas of PF and ST were larger, we took 2 mm long samples, 15 mm to the left Luer and 30 mm to the right Luer, from each location for further analysis.

**Table 1:** Hemodynamic properties in ST, DS, HS, PF.

	ST			DS			HS			PF		
	Min	Max	Mean	Min	Max	Mean	Min	Max	Mean	Min	Max	Mean
TAWSS^a^	1.1E-06	2.1E-06	1.5E-06	0.02	0.31	0.11	0.16	0.87	0.35	0.32	0.32	0.32
OSI	0.32	0.48	0.42	0.02	0.36	0.20	0.03	0.21	0.08	0.06	0.06	0.06
Log (RRT)^a^	14.15	16.90	15.32	1.26	4.26	2.75	0.24	2.35	1.31	1.25	1.27	1.26
TransWSS^a^	−17.58	−8.89	−12.23	−3.62	6.89	0.73	−3.33	5.02	1.99	−6.87	−1.28	−3.28
Reynolds number	0	0	0	0	10.07	4.82	0	39.58	20.13	0	38.61	18.64

a TAWSS, dyne/cm2; Log (RRT), 1/Pa; TransWSS, dyne/cm2.

### 3.3 High-content screening revealed the morphological diversity of HUVECs under different hemodynamics conditions

**Fig. 4:**
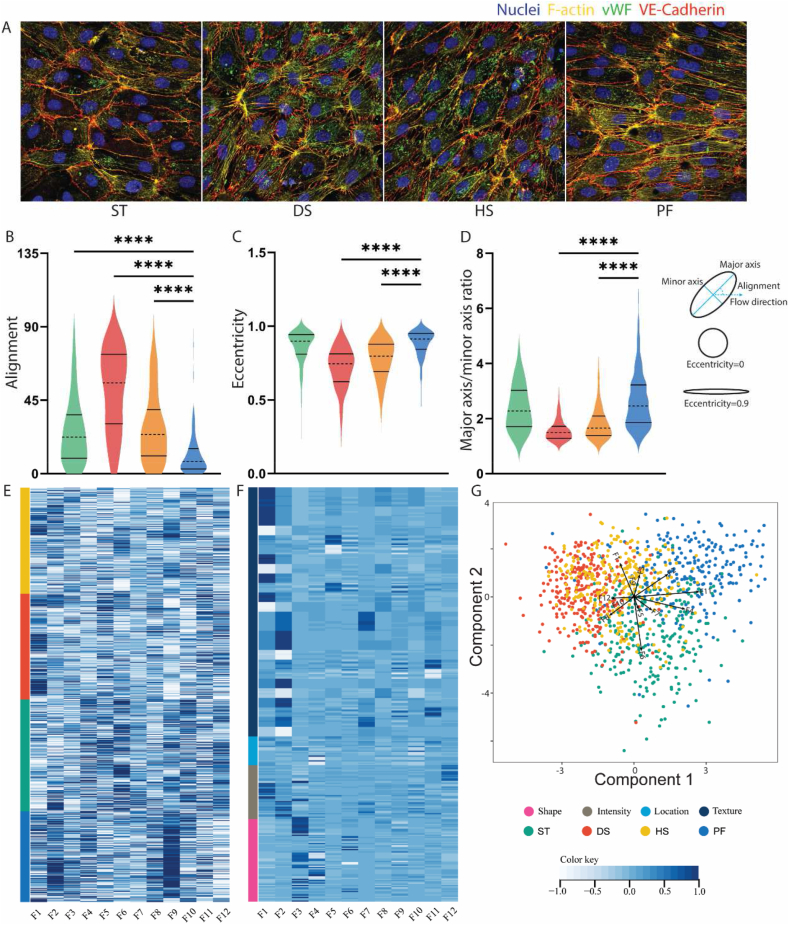
Cell morphology of HUVECs under different flow conditions. (A) fluorescence images of HUVECs in ST, DS, HS, PF. (B, C, D) Violin graph showing distribution of HUVECs alignment, eccentricity, ratio of major axis/minor axis in ST, DS, HS, PF. (E) Heatmap concluding the distribution of 12 discriminative factor in ST, DS, HS, PF. (F) Contribution of cellular compartment and feature class to each Factor. (G) Biplot of LDA model score and factor coefficients.

A total of 1050 cells (ST 283, DS 268, HS 269, PF 230) from three individual experiments were included in the morphological analysis. Cells displayed distinct visually identifiable differences between the different flow patterns, (Fig. 4A). Shape and flow alignment of the HUVECs were computed at single-cell resolution for the four CFD predicted locations. Cell alignment was defined as the absolute angle between the cell major axis and the flow direction. Cells experiencing PF were highly aligned with the flow, with a median alignment angle of 7.43° (IQR, 2.92°, 15.6°) (Fig. 4B). HUVECs experiencing DS displayed highly disorganized alignment, with a median angle of 55.67° (IQR, 30.82°, 73.07°). HS and ST showed marginal alignment with the direction of flow, with median angle 23.93° (IQR, 10.99°, 39.00°) for HS and median angle 22.36° (IQR, 9.52°, 35.94°) for ST (Fig. 4B). Elongation of the cells was quantified using both eccentricity and the ratio of major/minor axis [41]. For HUVECs experiencing PF, eccentricity median 0.91 (IQR, 0.85, 0.95), ratio median 2.46 (IQR, 1.88, 3.22) were significantly more elongated and elliptical compared to DS eccentricity median 0.74 (IQR, 0.62, 0.81), ratio median 1.50 (IQR, 1.28, 1.71) or HS eccentricity median 0.80 (IQR, 0.69, 0.88), ratio median 1.66 (IQR, 1.38, 2.09). Interestingly, ST with eccentricity median 0.90(IQR, 0.81, 0.94), ratio median 2.27 (IQR, 1.71, 3.02) did not show any different compared to PF (Fig. 4B and C).

To further stratify the different HUVEC populations in the four locations experiencing different hemodynamics, a modification of the Cell Painting assay was used to extract single-cell morphological profiles [42]. Heatmaps and UMAPs concluding overview features for 1050 cells are shown in supplement (Fig. S4). Single-cell morphological profiles containing 360 features were reduced in dimension by Factor Analysis, resulting in 12 Factors representing 81 % of total variance, among them, Factor 1 and 2 contribute 44.7 % of total variance (Fig. 4E).

Subsequently, supervised multivariate analysis of the reduced profiles using an LDA classification model showed separate HUVEC populations for the PF, DS, and ST hemodynamics locations. The HUVEC phenotypes associated with the HS location displayed overlap between the different locations, and likely resemble a more intermediate phenotype (Fig. 4F). To aid in biological interpretation of the model, model coefficients were visualized in the score plot as vectors for each factor. The vector direction was used to assess the contribution of each factor to the class separation, and the factors oriented perpendicular to the line separating two classes were regarded as important for that separation [34]. As a result, Factor 2, 8, 10, 11, 12 separated DS and PF; Factor 3, 6, 9 separated PF and ST; Factor 1, 5 separated DS and ST; Factor 4, 7 separated DS from PF and ST (Fig. 4G). When combined with the heatmap of factors and features (Fig. 4F), we can extrapolate that cell shape, intensity and texture of F-actin and VE-cadherin were the most discriminative features for separating DS from PF and ST, and cell shape was most discriminatory between PF and ST.

### 3.4 Cell adherens junction remodeled under disturbed flow

**Fig. 5:**
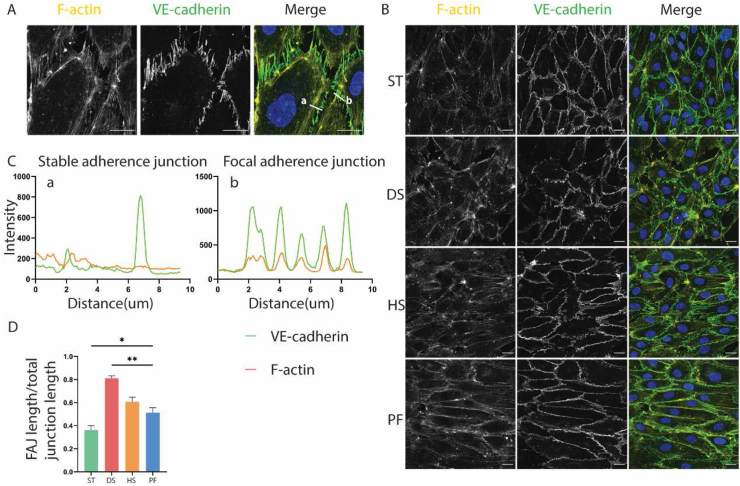
Cell adherens junction of HUVEC under flow. (A) fluorescence colocation revealed the overlap of VE-cadherin and F-actin in FAJs, while they did not overlap in stable AJs, scale bar = 20 μm. (B) Overview of cell adherens junctions of HUVEC in ST, DS, HS, PF, scale bar = 20 μm. (C) Colocation of VE-cadherin and F-actin in stable AJs and FAJs. (D) Ratio of FAJs length over total adherens junction length in ST, DS, HS compared with PF, n = 3.

After two days of flow, adherens junctions of HUVECs in DS showed a higher degree of remodeling than PF and other two areas. The remodeled adherens junctions induced by disturbed flow showed typical focal adherens junctions (FAJs) properties. Fluorescence colocation revealed the spatial overlap of VE-cadherin and F-actin in FAJs, and FAJs were aligned perpendicularly with adherens junctions. Conversely, F-actin and VE-cadherin did not overlap in stable AJs, and AJs ran parallel to F-actin bundles, (Fig. 5A–C). Fluorescence imaging revealed that F-actin oriented at the periphery of the cells and was shorter than that in HS and PF. The VE-cadherin signals were linear and continuous in PF, whereas in DS, they were discontinuous and stressed. The VE-cadherin appearance of HS was between PF and DS. Compared to the other three locations, the alignment of F-actin and VE-cadherin in ST was different, F-actin were also short and aligned randomly in ST, while VE-cadherin was thick and continuous, (Fig. 5B). The quantification of the ratio of FAJ length over the total junction length showed that it was significantly higher in DS than PF. Multiple comparisons showed that ST had less FAJs than DS and HS, but comparable with PF. The result showed no differences between the ratio in HS and PF (Fig. 5D). The results revealed that more adherens junctions were remodeled under disturbed flow than laminar flow.

### 3.5 Impact of disturbed flow on markers of EC function

**Fig. 6:**
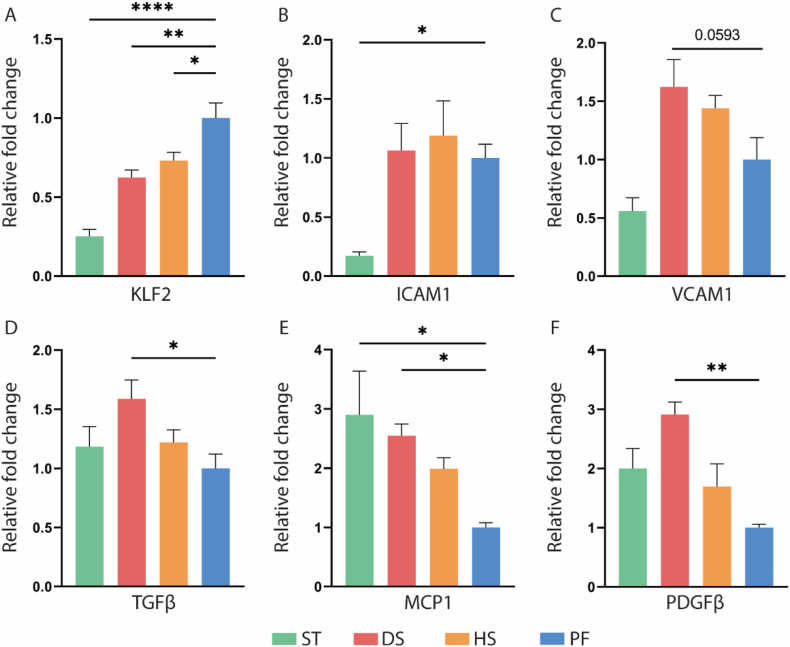
Comparison of relative gene expression from static (ST), disturbed flow (DS), high WSS (HS) regions with physiological flow (PF) region. (A) KLF2; (B)ICAM1; (C) VCAM1; (D) MCP1; (E) TGFβ; (F) PDGFβ. n = 4.

The effect of altered hemodynamics on HUVECs was assessed through measuring gene expression of various markers of EC function via RT-qPCR (Fig. 6). When compared to PF, the expression of the shear stress sensor KLF2 on the membrane of HUVECs was downregulated in DS and ST. Intercellular adhesion molecule 1 (ICAM1) expression in ST was much lower than in other areas, although there were no changes seen between DS and HS and PF. When comparing the vascular cell adhesion protein 1 (VCAM1) expression level across all sites to PF, no variations were observed. Conversely, chemoattractant protein 1 (MCP1) expression in ST and DS was three times greater than in PF, while there was no difference between HS and PF. Transforming growth factor β (TGFβ) expression in DS was also noticeably higher than in PF, while other locations did not show any differences when compared to PF. Platelet-derived growth factors β (PDGFβ) expression was more than three times higher in the HUVECs exposed to disturbed flow than in the PF region. However, ST, HS, and PF did not exhibit any changes.

## 4 Discussion

In this study, a standardized and modularized *in vitro* model was produced that mimics disturbed flow in human vascular system. The applicability of the model was assessed by examining the morphological and functional diversity of HUVECs under different hemodynamics conditions (stagnant condition, disturbed flow, high WSS, physiological flow) in the same model. The results showed that this model can be used as a platform for future research on IH. Furthermore, this model can be employed to evaluate the therapeutic value of new drugs designed to impede these pathological processes.

### 4.1 Integration of in vivo branching structure and stimuli in one *in vitro* bypass flow model

*In vitro* vascular disease models require multiple properties to simulate native vascular system, like 3D structure, 3D cell culture, cell-cell interactions, mechanical stimulation [43]. The conventional models, including cone-and-plate as well as microfluidics devices, cannot meet the requirement, so innovative *in vitro* models are necessary. 3D bio-printing is a revolutionary tissue engineering technique that can print cells and extracellular matrix directly into millimeter-scale 3D vascular systems, more closely mimicking physiological conditions. But producing branching vessels is still challenge, and excessive costs limits 3D bio-printing accessibility [44]. To overcome the limitation of previous *in vitro* vascular model and increase application scenarios, the bypass flow model presented here contains several design highlights.

1. The model is a branching 3D structure mimicking the configuration and dimensions of vascular bypass grafts, where IH is common. The channel diameter is 2.5 mm, which is close to the native vessel size. After introducing flow to the channels, this model can establish more complex hemodynamics compared to submillimeter microfluidic devices allowing to study physiological or pathological shear stress in a single model.
2. The cell culture substrate is compliant and biological relevant. Various biocompatible matrixes are suitable for the bypass flow model. Furthermore, the hydrogel with a thickness of 1 mm offers space for potential 3D co-culture of smooth muscle cells and EC. It is also worth noting the amount of collagen used, 300ul, is far smaller than the size of the entire model, which significantly lowers expenses.
3. The modular design allows to continuously upgrade and modify of each part of the model. For example, by adding a helical ridge to the branch part's channel, the inlet flow pattern may be altered to suit various research objectives [45,46]. Additionally, the detachable structure makes it simple to remove collagen and cells after an experiment without damaging the model, which makes it easier to conduct follow-up tests and treatments.
4. Plastic injection technology has been used to manufacture hundreds of models for this study, which significantly cut costs and manufacturing times while maintaining accuracy between each model. In addition to preventing errors in CFD simulation and enhancing the reliability of CFD results, the standardized model also enhances comparability and repeatability with other studies that employ same models.
5. The leak-free Luer connectors in the model are also utilized in most medical and experimental fluid items. The three-way design allows us to flexibly adjust the inlet and outlet location, different blood flow directions can be simulated in this model for expanding the applicability [47].

### 4.2 Induction of four specific hemodynamics flow patterns in one channel

CFD predicted the locations of disturbed flow as well as other regions with various hemodynamic features in this bypass model. The vessel bifurcation and the vascular bypass graft both induce intense disturbed flow in the intersection area [1,2]. The latter configuration was chosen for this study because a straight host vessel was more practical for cell culturing and handling. In addition, the floor of the host vessel is the location where IH prefers developing [48,49]. Previous CFD showed the typical hemodynamics of disturbed flow here, where TAWSS were low and OSI, RRT, TransWSS were rising due to the sharp change and pulsatile flow pattern in the vascular structure [45]. The CFD results of DS in the model showed similar hemodynamic distributions as other in other studies. The velocity plot indicated that the flow was reattached and separated. The hemodynamics of the HS area shift dramatically where the TWASS was the greatest of the four locations, while the OSI and RRT were low, interestingly, the value of TransWSS was still high in this location, which indicated the multidirectional flow here, and it was still possible to see eddies in this location at the end of the peak flow. The hemodynamics in PF performed with uniform TAWSS and low OSI, RRT, and the velocity vector was theoretically unidirectional. However, the flow direction might have an instantaneous axial oscillation based on the pressure waveform captured from the inlet. It underlines that the flow in ST area was stagnant, and the flow velocity, WSS, TransWSS tended to be zero, but the high OSI was influenced by the pulsatile pressure from the Ibidi pump. Therefore, four locations with typical hemodynamic features were divided in the model, and subsequent validations were handled in these locations.

### 4.3 The flow in the bypass model altered HUVEC morphology and adherens junction

The alignment and elongation of ECs in the vessel are considered as morphological features of vascular homeostasis. The response of cytoskeleton and cell junctions to the flow contribute to the homeostasis jointly [50]. In physiological conditions, the junctional mechanosensory complex, made up of VEGFR2, PECAM-1, and VE-cadherin, is static. ECs are elongated, and their alignment are along the flow direction. EC junctions are stable and are aligned by thick parallel actin which do not overlap with VE-cadherin. The physiological cell morphology and junction preserve the permeability and integrity of the cell barrier function which plays a critical role in protecting against IH. Currently, the disturbed flow represented by oscillatory and low shear stress is considered as the major factor leading to initiate and develop vascular disease [51,52]. The junctional mechanosensory complexes become active and begin to transduce mechanical stimuli into cells when the flow is disturbed. In this case, actomyosin tension rises and starts to pull F-actin, a cytoskeletal component. As a result, stable AJs start converting to FAJs and causing cell-cell adhesion remodeling, junctions become discontinuous. Meanwhile, the shape of cells turns round, actin turns short and disperses randomly at the cell periphery. Ultimately, the cell barrier is dysfunctional, and permeability increases [11,53].

In our study, we compared the morphology and junctions of HUVECS in DS with other locations to confirm the validity of disturbed flow induced by our model. High-content screening was used to catch single-cell morphological features from four areas, Biplot after LDA analysis showed the contribution of morphological features to the classification and revealed that the disturbed flow could affect HUVECs cytoskeleton organization in DS when compared to other locations. Confocal microscopy confirmed that F-actin bundled and orientated perpendicularly to VE-cadherin and formed FAJs. The higher ratio of FAJs indicated that disturbed flow weakens the cell-cell junction and cell barrier function. These changes of morphology and junctions in DS were comparable with previous studies, mainly related to the low WSS and high OSI [11,53,54]. The ST had the highest OSI and RRT but lowest TAWSS and TransWSS in the model. However opposite to the cells in DS and PF, the HUVECs in the stagnant condition were similar as the cell morphology in static condition [39]. These interesting results seem to contradict the effect of disturbed flow. The velocity and WSS videos confirmed that the fluid was likely static and maintained almost 0 cm/s in the whole pump cycle, even though the high OSI was probably caused by the pulsatile pressure from the pump. Future studies should confirm the lower limit of WSS sensitivity in ECs and the reaction of ECs to the extremely low WSS. Another interesting finding was that the HUVECs in HS did not show significant alignment and elongation as PF even if the OSI, RRT in HS were low and TAWSS was high. The morphology of HUVECs in HS and DS was more alike than HS and PF, the potential reasons could be the distance to the DS was so close that the flow did not fully develop, and the multi-directionality of flow also effected the cell morphology as the TransWSS was high in HS [10].

### 4.4 Disturbed flow induced IH related markers of HUVECs

The switching from a healthy phenotype of ECs to inflammatory phenotype is the initiation IH [55]. Disturbed flow is a main mechanical factor inducing this shift. Under physiological flow pattern, KLF2 is a crucial mechanosensitive transcription factor that regulates EC inflammation by blocking the expression of ICAM1, VCAM1, and MCP1 [56]. KLF2 also down-regulates the expression of TGFβ to maintain quiescent endothelial phenotype by inhibiting endothelial-to-mesenchymal transition which participates in the pathogenesis of IH. Conversely, in disturbed flow, KLF2 expression is inhibited and TGFβ and inflammatory markers are elevated. Furthermore, PDGFβ is also increased in disturbed flow. Vascular smooth muscle cells are known to be stimulated by PDGFβ to change from a contractile phenotype to a proliferative one and migrate from vessel media to intima, together with other immune cells recruited by inflammatory cytokines, developing of hyperplasia [17,[57], [58], [59]]. The induction of IH related makers in HUVECs upon exposure to disturbed flow conditions is in line with previous studies. the HUVECs under disturbed flow expressed more MCP1, TGFβ, and PDGFβ and less KLF2 when compared to the laminar flow pattern. However, ICAM1 and VCAM1 did not vary in either of these two sites. This was due to the fact that our flow experiment lasted for two days, while after one day, the expression levels of VCAM1 and ICAM1 most likely returned to normal which were aligned with the previously published RNA-seq data [24,25].

## 5 Study limitations

The pulsatile IBIDI system flow was a tooth-like pattern and deviated from a natural blood flow pattern. In addition, the area and number of HUVECs affected by disturbed flow in our study remained small, and the low protein level limited the use of traditional protein quantification techniques, even though a wider channel was used than in microfluid chips. Lastly, by assuming a rigid wall model, CFD was utilized to predict the disturbed flow location, but collagen is an inherently soft matrix which might distort under flow and affect the accuracy of prediction requiring the use of more complicated CFD techniques such as fluid-structure interaction (FSI) to capture its dynamics. The flow rate in our model was still far smaller than the flow rate in native bypass grafts or vessels, this was limited by the pump and the matrix stiffness we used. The duration of our flow experiment was 2 days, while it took more time to develop IH in native vascular system, longer duration should be studied in the future.

## 6 Conclusion and prospection

In this study, we created an *in vitro* model that could induce pathological flow at a specific location. We compared morphology and cell-cell junction of HUVECs, and IH related markers in the intersection area, whereas the areas with other flow patterns are used to verify the validity of our model. The findings demonstrate that this model is a useful platform to investigate the causal relation between pathological flow patterns and EC dysfunction.

Endothelial cells are continuously subjected to a complex mechanical environment in the human body. Mechanical factors like WSS, cyclic stretch and pressure are attributed to the blood flow [60], and other factors like stiffness, curvature, and topology are attributed to extracellular matrix [61]. Abnormal range of these mechanical factors can cause EC dysfunction and further induce vascular disease. Combining several mechanical factors into one model is challenging due to limitations of the technology and materials. In our future study, we aimed to overcome this limitation by optimizing the flow settings by increasing the flow volume and pressure to a native range, and also adjusting the stiffness of matrix to mimic physiological and diseased vessels. The mature model should be able to replicate different conditions of blood vessels, advancing the field of biological research and medication development.

## CRediT authorship contribution statement

**Zhuotao Xiao:** Writing – original draft, Visualization, Validation, Methodology, Funding acquisition, Formal analysis, Data curation, Conceptualization. **Rudmer J. Postma:** Writing – review & editing, Visualization, Validation, Software, Formal analysis. **Anton Jan van Zonneveld:** Writing – review & editing, Resources. **Bernard M. van den Berg:** Writing – review & editing, Resources. **Wendy M.P.J. Sol:** Writing – review & editing, Resources. **Nicholas A. White:** Writing – review & editing, Resources. **Huybert J.F. van de Stadt:** Resources. **Asad Mirza:** Writing – review & editing, Visualization, Validation, Software, Formal analysis. **Jun Wen:** Writing – review & editing, Visualization, Validation, Software, Formal analysis, Conceptualization. **Roel Bijkerk:** Writing – review & editing, Supervision, Conceptualization. **Joris I. Rotmans:** Writing – review & editing, Supervision, Resources, Project administration, Funding acquisition, Conceptualization.

## Declaration of competing interest

The authors declare that they have no known competing financial interests or personal relationships that could have appeared to influence the work reported in this paper.

## Data Availability

Data will be made available on request.
