# Supplementary material for: A bypass flow model to study endothelial cell mechanotransduction across diverse flow environments
Source: Mater Today Bio. 2024 Jun 13;27:101121. doi: 10.1016/j.mtbio.2024.101121 (PMC11234155; doi:10.1016/j.mtbio.2024.101121)
Supplement: Multimedia component 1 [file mmc1.docx]

**A Bifurcated Flow Model to Study Endothelial Mechanotransduction Across Diverse Flow Environments**

Supplemental material

Zhuotao Xiao, Rudmer Postma, Anton Jan van Zonneveld, Bernard M. van den Berg, Wendy Sol, Nicholas A. White, Huybert van de Stadt, Asad Mirza, Jun Wen, Roel Bijkerk, Joris I. Rotmans


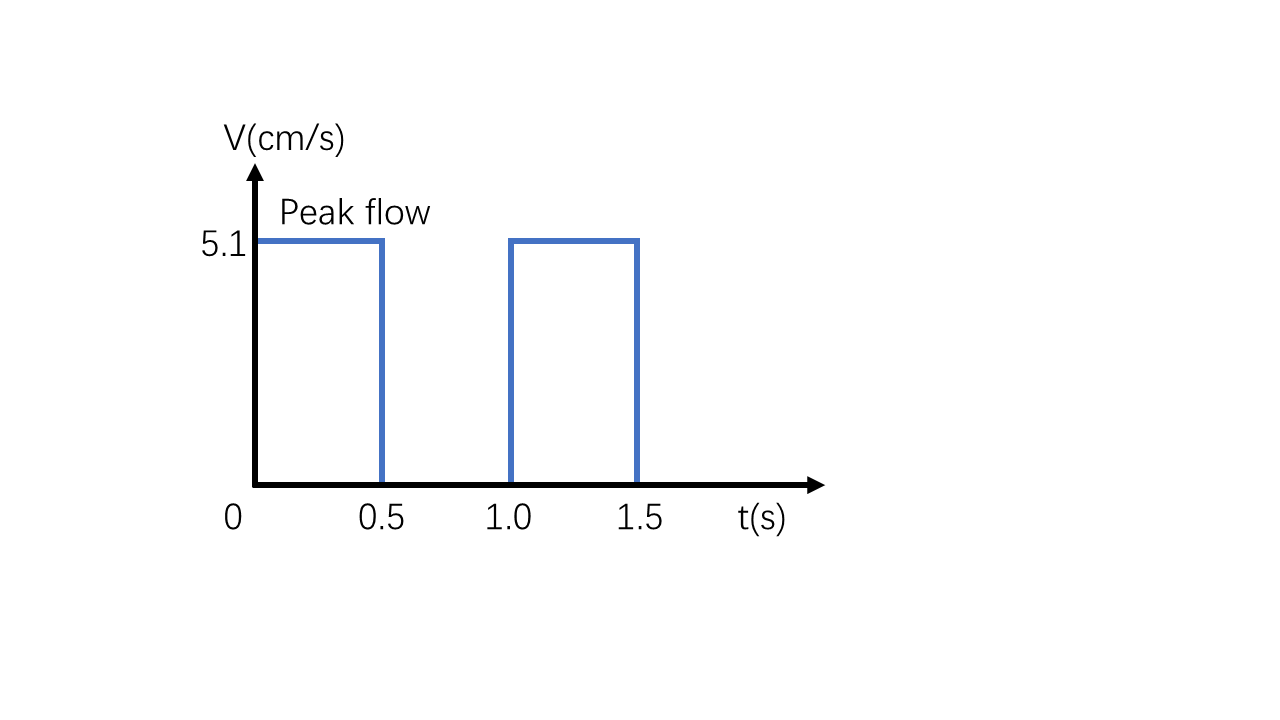
A


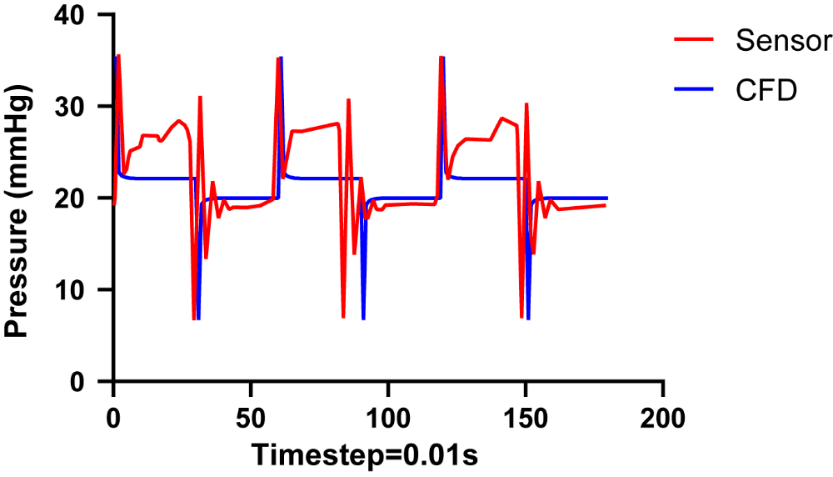
B

Fig.S1: (A) flow pattern pumped to the inlet from Ibidi system. (B) Inlet pressure waveform measured by pressure sensor and inlet pressure waveform recorded by CFD after calibration.


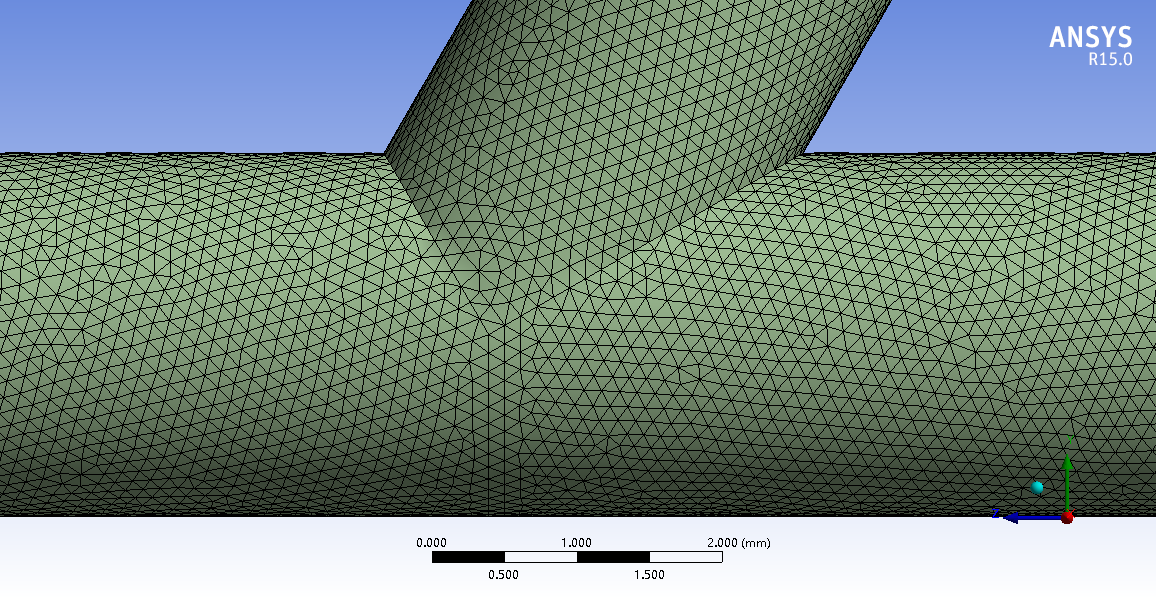


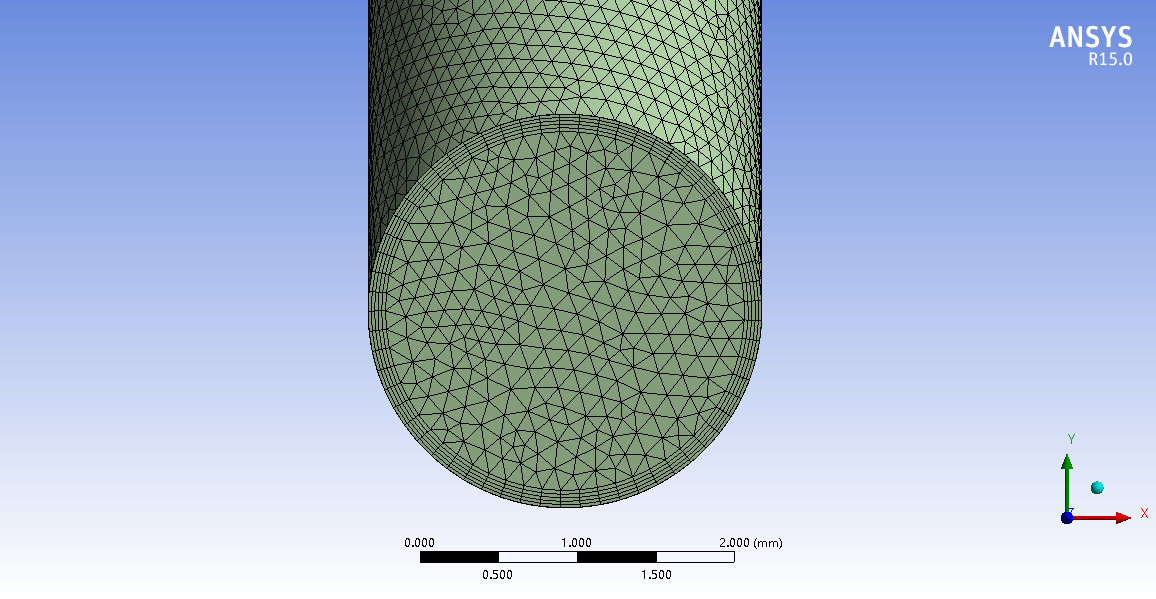
Fig.S2: Mesh for CFD simulation.


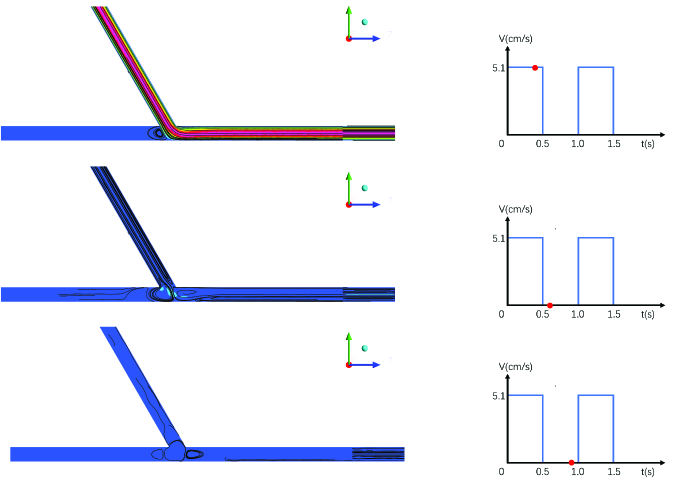


Fig.S3: Streamline showed the direction of the flow in disturbed flow model at time point t1=0.45, t2=0.55, t3=0.95.


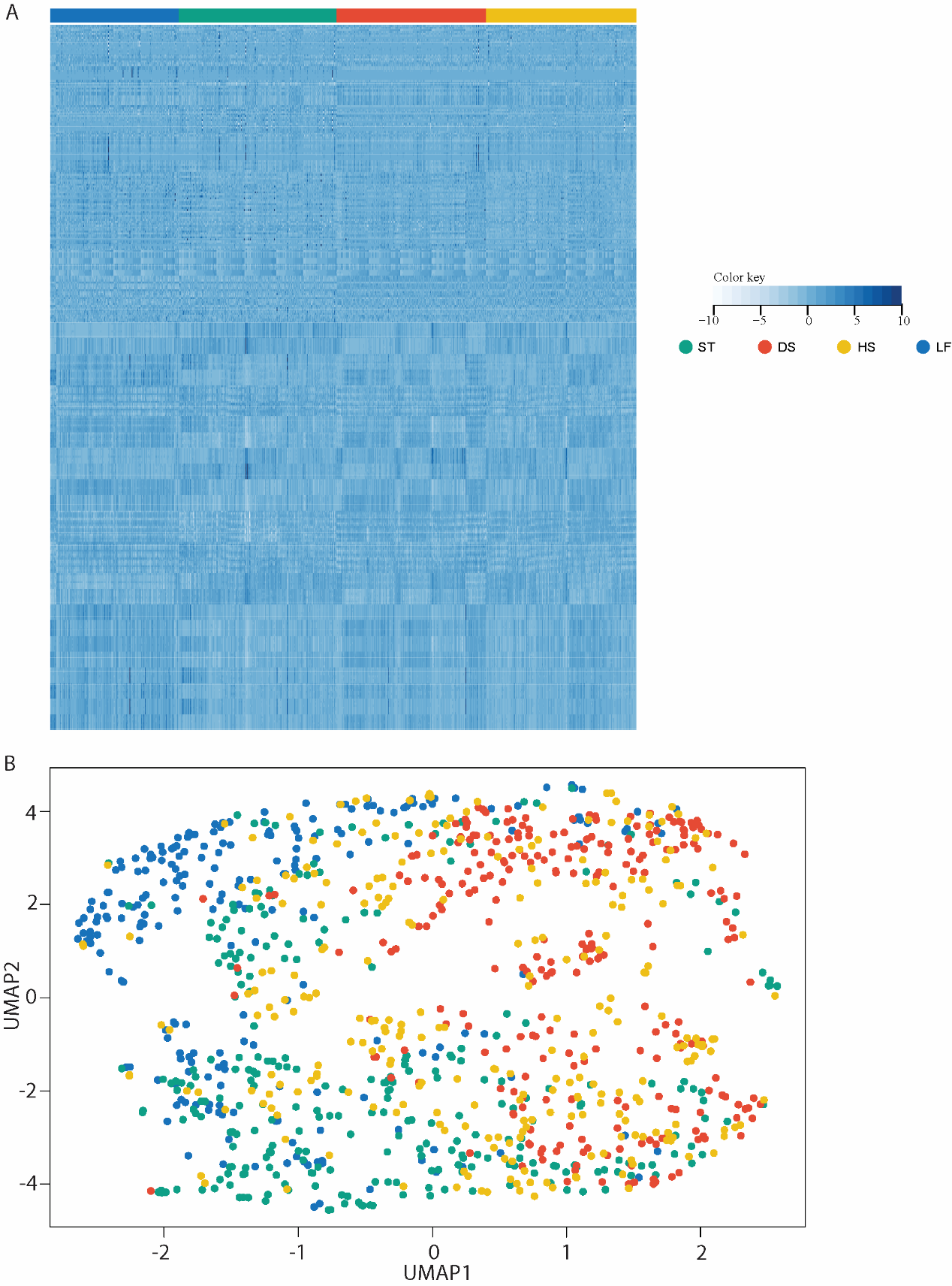


Fig.S4: (A) heatmap of 1050 cells from ST, DS, HS, ST showing properties of 360 morphological features. (B) UMAP reduce dataset dimension and show the distribution of HUVECs on the plot based on morphological features.

Table S1: mesh sensitivity analysis

| Mesh | Maximum Cell Length (mm) | Number of Elements | Max Wall Shear Stress (AWSS) | | Average Wall Shear Stress (AWSS) | |
| --- | --- | --- | --- | --- | --- | --- |
|  |  |  | Max WSS (dynes/cm2) | Percent Diff (%) | AWSS (dynes/cm2) | Percent Diff (%) |
| 1 | 0.205 | 504925 | 1.297014 | 28.5% | 0.379959 | 0.2% |
| 2 | 0.17 | 816720 | 1.510765 | 16.8% | 0.379633 | 0.1% |
| 3 | 0.14 | 1263398 | 1.683923 | 8.9% | 0.378242 | 0.6% |
| 4 | 0.128 | 1595107 | 1.745913 | 3.9% | 0.379583 | 0.3% |
| 5 | 0.117 | 1986742 | 1.805875 | 0.5% | 0.378614 | 0.5% |
| 6(Ref) | 0.107 | 2480218 | 1.81539 | 0% | 0.380602 | 0% |

Table S2: time-step independence analysis

| Time-step | Max Wall Shear Stress (AWSS) | | Average Wall Shear Stress (AWSS) | |
| --- | --- | --- | --- | --- |
|  | Max WSS (dynes/cm2) | Percent Diff (%) | AWSS (dynes/cm2) | Percent Diff (%) |
| 0.1 | 1.6839229 | 4.7% | 0.37824216 | 5% |
| 0.01 | 1.6053287 | 0.13% | 0.36186 | 0.5% |
| 0.001(Ref) | 1.60739 | 0% | 0.36017 | 0% |

Table S3 Primers used for q-PCR

| Gene | Forward 5’-3’ | Reverse 5’-3’ |
| --- | --- | --- |
| GAPDH | TTCCAGGAGCGAGATCCCT | CACCCATGACGAACATGGG |
| KLF2 | CTACACCAAGAGTTCGCATCTG | CCGTGTGCTTTCGGTAGTG |
| ICAM1 | GTATGAACTGAGCAATGTGCAAG | GTTCCACCCGTTCTGGAGTC |
| VCAM1 | TTTGACAGGCTGGAGATAGACT | TCAATGTGTAATTTAGCTCGGCA |
| TGFβ | CTGCCCCTACATTTGGAGC | AGCGCACGATCATGTTGGAC |
| MCP1 | CCAGCAGCAAGTGTCCCAAAG | TGCTTGTCCAGGTGGTCCATG |
| PDGFβ | CTCGATCCGCTCCTTTGATGA | CGTTGGTGCGGTCTATGAG |
